# Psychoemotional Profiles in Reading Comprehension Among Students with Typical Development, Learning Disabilities, and Developmental Language Disorder

**DOI:** 10.3390/bs16050759

**Published:** 2026-05-13

**Authors:** Diamanto Filippatou, Panagiota Dimitropoulou, Elisavet Chrysochoou, Asimina M. Ralli

**Affiliations:** 1 Department of Psychology, National and Kapodistrian University of Athens, 15784 Athens, Greece; asralli@psych.uoa.gr; 2 Department of Psychology, University of Crete, 74150 Rethymno, Greece; p.dimitropoulou@uoc.gr; 3 School of Psychology, Aristotle University of Thessaloniki, 54124 Thessaloniki, Greece; echrysoc@psy.auth.gr

**Keywords:** psychoemotional profiles, reading comprehension, learning disabilities, typical development, developmental language disorder, third grade students

## Abstract

The present study examined psychoemotional profiles associated with reading comprehension among third-grade Greek students with typical development, learning disabilities, and developmental language disorder. A person-centered approach was used to identify distinct profiles based on academic emotions, reading motivation, and reading comprehension performance. The sample consisted of 83 third-grade students from public elementary schools in Attica, Greece (mean age = 107.45 months). Participants were classified into three groups: typically developing students, students with learning disabilities, and students with developmental language disorder. Hierarchical cluster analysis using Ward’s method followed by k-means clustering was conducted separately for each group. Two psychoemotional profiles emerged in all three groups. In the typically developing and learning disabilities groups, the profiles differed in emotional and motivational characteristics but not in reading comprehension performance. In contrast, in the developmental language disorder group, the profiles differed significantly in reading comprehension: one profile was characterized by lower comprehension, higher negative emotions, and higher motivation, whereas the other showed higher comprehension, more positive emotions, and lower motivation. These findings highlight the heterogeneity of psychoemotional experiences associated with reading and suggest that the role of reading comprehension in profile differentiation may vary across developmental groups. The results underscore the importance of addressing both cognitive and psychoemotional aspects of reading in educational interventions, particularly for students with developmental language disorder.

## 1. Introduction

Reading comprehension is essential for academic success, cognitive development, career advancement, and everyday life. Its importance is even greater for students with learning disabilities and developmental language disorder, as persistent difficulties in comprehension can limit broader academic progress and lifelong learning.

Even typically developing (TD) students may experience comprehension difficulties at early stages of reading development, when decoding demands limit the cognitive resources available for higher-order tasks such as inference making (Bonifacci et al., 2022; van Zeijts et al., 2024).

For students with LD, who experience persistent difficulties in word reading and phonological processing, reading comprehension problems often extend to higher-order processes such as text processing and inference making (Lievore et al., 2025; Nachshon & Horowitz-Kraus, 2019; Polychroni et al., 2024). Similarly, students with DLD experience comprehension difficulties due to broader linguistic constraints, including limited vocabulary, weaknesses in syntactic processing, and working memory constraints that interfere with building coherence across sentences (Nachshon & Horowitz-Kraus, 2019; Kritsotakis & Morfidi, 2024; Montgomery et al., 2021).

Beyond cognitive factors, reading outcomes are also shaped by interrelated emotional and motivational processes (Zaccoletti et al., 2020). Reading difficulties may trigger anxiety, frustration, and avoidance, which can undermine motivation and further compromise comprehension (Fishstrom et al., 2024; Mano et al., 2017; Zaccoletti et al., 2020).

More specifically, children with LD are at increased risk for anxiety, low self-esteem, negative academic self-concept, depression, and broader socioemotional difficulties (Adi et al., 2024; Ferreira et al., 2023; Mano et al., 2017; Nachshon & Horowitz-Kraus, 2019; Zuppardo et al., 2023). In parallel, children with DLD show associations between linguistic difficulties and emotional regulation, which may further affect reading development (Aguilera et al., 2021; Burnley et al., 2023; Goh et al., 2021; Wenren et al., 2026). These bidirectional links between comprehension and emotional functioning highlight the complexity of reading development in these populations (Wenren et al., 2026).

However, most of this evidence relies on variable-centered group comparisons, which may obscure meaningful within-group variability. Such approaches can produce misleading implications when complex cognitive, emotional, and motivational processes are examined in isolation (Tetzlaff et al., 2023).

Cluster analysis and related person-centered methods address this limitation by identifying profiles based on patterns of co-occurring characteristics (Brandenburg et al., 2021; Miller-Cotto & Byrnes, 2026; Nyman et al., 2025; Tetzlaff et al., 2023). Although such approaches remain limited in research on LD and DLD, they offer particular promise for capturing psychoemotional heterogeneity.

Importantly, this type of research remains scarce, particularly in Greek educational contexts, which are still comparatively understudied in this field.

Identifying subgroups with shared cognitive–emotional patterns allows for the definition of profiles that may cut across diagnostic categories. This has clear implications for designing targeted, profile-sensitive interventions rather than relying solely on diagnostic categories (Brandenburg et al., 2021; Tetzlaff et al., 2023). 

### 1.1. Reading Comprehension as a Multifaceted Skill

Reading is a multifaceted cognitive skill that extends beyond decoding to include higher-order processes such as inference-making, integration of textual information with prior knowledge, and the construction of a coherent situation model (Bonifacci et al., 2022; van Zeijts et al., 2024). Although the Simple View of Reading conceptualizes reading comprehension as the product of decoding and listening comprehension (Gough & Tunmer, 1986), contemporary frameworks, particularly the Construction-Integration Model (Kintsch, 1988; Kintsch & Van Dijk, 1978), offer a more comprehensive account by describing comprehension across lexical, propositional, and situation levels of representation (Bonifacci et al., 2022; Sugawara et al., 2021). Within this model, readers first construct a network of ideas from the text and then integrate these with prior knowledge to form a coherent mental representation, a process that depends heavily on coherence and elaborative inferences (Barth et al., 2021; Zaccoletti et al., 2020).

Because these higher-order processes are highly sensitive to cognitive demands, beginning readers may struggle with comprehension when decoding is not yet automatized, leaving fewer resources available for inference-making and integration (Hsu et al., 2023; Juhkam & Soodla, 2022; Martins-Reis et al., 2023; Psyridou et al., 2021, 2023; van Zeijts et al., 2024). Such difficulties are particularly evident in students with dyslexia and developmental language disorder (DLD), who often experience deficits in generating the inferences necessary for meaning construction.

Students with reading comprehension difficulties often struggle to generate both coherence and elaborative inferences (Ferreirós et al., 2021). These difficulties are particularly evident in students with learning disabilities (e.g., dyslexia) and in those with developmental language disorder (DLD), who often struggle to produce the inferences required to construct meaning from text (Freed & Cain, 2021; Karasinski & Weismer, 2010; Kida et al., 2016; Kritsotakis & Morfidi, 2024). For example, children with dyslexia perform significantly more poorly on inferential than on literal comprehension questions, even when decoding is relatively intact (Lee et al., 2023). Similarly, students with DLD tend to use fewer cohesive devices, resulting in ambiguity in their narratives and hindering the integration of textual information necessary for inference making (Ahufinger et al., 2025; Archibald, 2024; Delgado-Cruz et al., 2021; Wong et al., 2022). Together, these findings highlight the particular vulnerability of higher-order comprehension processes in neurodevelopmental groups, especially when cognitive resources are constrained by basic decoding demands (Baek & Yim, 2022; Capin et al., 2021; Le Cunff et al., 2024; van Zeijts et al., 2024).

### 1.2. Emotions and Reading Comprehension

Psychoemotional factors play a central role in reading comprehension by shaping not only students’ affective experiences of reading but also the efficiency with which they process and understand text. Research over the past decades has shown that reading is not exclusively a cognitive activity; rather, it is also influenced by academic emotions such as enjoyment, anxiety, and boredom (Rogiers et al., 2019; Pekrun et al., 2002; Prinz-Weiß et al., 2023; Zaccoletti et al., 2020). These emotions arise from students’ perceptions of control over reading tasks and the value they assign to them within literacy contexts (Wang et al., 2022; Zaccoletti et al., 2020).

Within this framework, the control–value theory of academic emotions (Pekrun, 2000, 2019; Pekrun et al., 2002) offers a well-established account of how emotions operate in educational settings. It draws on expectancy-value perspectives on emotion, attribution theories of achievement, and broader models of emotion and achievement (Fredrickson, 2004; Pekrun, 1992; Turner & Schallert, 2001; Weiner, 1985; Zeidner, 2007). It proposes that students’ emotional responses are shaped by the degree of control they perceive over academic tasks and the value they attribute to those tasks, with important consequences for motivation and learning outcomes (Pekrun, 2024; Safferthal & Prinz-Weiß, 2025). Different categories of emotions are assumed to exert distinct functional effects: activating positive emotions, such as enjoyment and pride, generally support sustained attention, cognitive flexibility, and efficient use of resources, whereas deactivating negative emotions, such as boredom and hopelessness, tend to impair performance by encouraging superficial information processing (Pekrun, 2024; Safferthal & Prinz-Weiß, 2025; Zhang et al., 2024).

Accordingly, when students perceive themselves as competent and view reading tasks as meaningful, they are more likely to experience positive emotions such as enjoyment and pride (Prinz-Weiß et al., 2023; Safferthal & Prinz-Weiß, 2025). These activating positive emotions have been associated with greater attention, persistence, and flexible strategy use (Pekrun et al., 2002; Pekrun & Perry, 2014). In reading contexts, enjoyment appears to facilitate deeper processing, stronger integration of ideas, and more effective self-regulation, including goal setting, comprehension monitoring, and strategic adjustment when understanding breaks down (Dimitropoulou et al., 2025).

By contrast, when students perceive low control, particularly in relation to tasks they consider important, negative emotions such as anxiety, shame, and frustration are more likely to emerge (Jiménez-Pérez et al., 2023; Prinz-Weiß et al., 2023; Zaccoletti et al., 2020). These activating negative emotions may consume cognitive resources through worry and self-doubt (Xie et al., 2026). Although they can occasionally increase effort through avoidance-based motivation, they are more often associated with rigid or surface-level strategies, such as memorization and rehearsal, rather than deep comprehension (Barnes et al., 2023; Pekrun et al., 2002, 2023).

Positive deactivating emotions, such as relief or relaxation, may also reduce effort temporarily and thereby encourage more superficial processing. Negative deactivating emotions, especially boredom and hopelessness, are particularly detrimental to reading comprehension because they undermine attention, reduce persistence, and promote shallow engagement with text (Pekrun et al., 2002; Pekrun & Perry, 2014; Zhang et al., 2024). Over time, repeated experiences of such emotions may weaken intrinsic motivation to read, reduce reading practice, and ultimately increase the effort required for successful comprehension (Ghaderi et al., 2022; Turnquest et al., 2024; Zaccoletti et al., 2020).

### 1.3. Motivation and Reading

Motivational factors play a critical role in academic achievement, particularly in reading comprehension. Within Self-Determination Theory (SDT), motivation is conceptualized along a continuum ranging from controlled to autonomous regulation (Ammel et al., 2021). Intrinsic motivation refers to engagement in an activity for its inherent satisfaction, enjoyment, or personal interest (Wigfield & Guthrie, 1997). In reading, this includes reading for pleasure, curiosity, learning, or the challenge involved in understanding a text. Extrinsic motivation, by contrast, involves engagement in reading to attain outcomes separate from the activity itself, ranging from external pressures, such as grades or avoidance of punishment, to more internalized forms in which the reader values the outcome even if the activity is not inherently enjoyable (Ammel et al., 2021; De Naeghel et al., 2012; Wigfield & Guthrie, 1997).

Within this framework, motivation ranges from amotivation to controlled and autonomous forms (Vuorinen et al., 2024). Autonomous motivation, in particular, is characterized by personal choice and self-endorsement, reflecting alignment with one’s interests and values (Villalobos-Zúñiga et al., 2021). SDT further posits that the fulfillment of three basic psychological needs—autonomy, competence, and relatedness—supports motivation and promotes adaptive functioning (De Naeghel et al., 2012; De Smedt et al., 2020). When these needs are satisfied in educational settings, students are more likely to display stronger motivation and, consequently, higher reading achievement (De Smedt et al., 2020; De Naeghel et al., 2012).

A complementary sociocultural perspective emphasizes that motivation is shaped by social, cultural, and contextual influences rather than emerging solely from individual dispositions (Wigfield & Koenka, 2020). From this perspective, students’ interactions with teachers, peers, family members, and the broader sociocultural environment contribute to the formation of their goals, values, and beliefs about academic engagement.

Empirical evidence consistently demonstrates a positive association between reading motivation, particularly autonomous forms, and reading comprehension outcomes (Ammel et al., 2021; Dimitropoulou et al., 2021; Dimitropoulou et al., 2025; Heyne et al., 2023; Jang & Conradi Smith, 2026; Vuorinen et al., 2024). Topic interest and reading amount, both closely linked to intrinsic motivation, have been shown to enhance comprehension by increasing engagement with text, especially among students with lower reading achievement (Bråten et al., 2022; Rachman, 2018; Troyer et al., 2019). This relationship appears to be reciprocal, such that motivation supports comprehension, while successful comprehension, in turn, strengthens motivation (Iaia et al., 2024; Ives et al., 2023).

By contrast, controlled motivation, driven primarily by external demands, does not appear to have the same positive association with reading ability (Ammel et al., 2021; Vuorinen et al., 2024). Indeed, excessive reliance on extrinsic incentives may undermine intrinsic motivation (Henning & Crow, 2023). When students read primarily for rewards or grades, they may lose interest in reading as a meaningful activity in itself; as a result, in the absence of external incentives, effort may decline, and text processing may become more superficial. Taken together, these findings suggest that the quality of students’ motivation, rather than its quantity alone, is particularly important for reading comprehension (Wei et al., 2020).

### 1.4. Neurodevelopmental Disorders and Reading Comprehension

#### 1.4.1. Students with Learning Disabilities (Including Dyslexia)

Students with learning disabilities (LD), particularly those with dyslexia, face distinctive psychoemotional challenges that can adversely affect reading comprehension. Dyslexia, characterized by persistent difficulties in word recognition and phonological processing, imposes not only cognitive demands but also increased psychological vulnerability relative to typically developing (TD) peers. Recent research consistently indicates that children with LD and dyslexia report lower reading motivation and academic self-concept, greater difficulties in emotional regulation, higher levels of anxiety, and lower trait emotional intelligence than TD students (Bonuomo et al., 2023; Gibby-Leversuch et al., 2019; Giovagnoli et al., 2020; Iaia et al., 2024; Livingston et al., 2018; Nachshon & Horowitz-Kraus, 2019; Niolaki et al., 2025; Polychroni et al., 2024; Sideridis, 2005, 2006; Sideridis et al., 2013, 2016; Wilmot et al., 2024; Zaccoletti et al., 2020).

Importantly, recent person-centered research further suggests that such psychoemotional and motivational characteristics do not form a uniform pattern across struggling learners; rather, students tend to cluster into distinct profiles that reflect different combinations of cognitive, motivational, and emotional strengths and vulnerabilities. For example, Miller-Cotto and Byrnes (2026) identified five relatively stable learner profiles based on prior reading achievement, motivation, and working memory, showing that these profiles significantly predicted later academic growth and cut across socioeconomic and diagnostic categories. Similarly, Nyman et al. (2025) found that students with and without special educational needs were represented across multiple motivational profiles, although students with SEN were more likely to belong to maladaptive profiles associated with lower performance and reduced time on task. Moreover, although the above studies highlighted variability in cognitive and motivational functioning, they did not specifically integrate emotional variables into the profiling of reading-related outcomes.

Extending this line of research, the present study examines reading-specific psychoemotional–motivational profiles in relation to reading comprehension across children with typical development (TD), learning disabilities (LD), and developmental language disorder (DLD). This approach addresses the limited attention previously given to diagnostic group differences, reading-specific emotional processes, and the Greek educational context.

These emotional and motivational difficulties, which may be further exacerbated by stigma and social pressures, can contribute to a maladaptive cycle in which reading difficulties evoke anxiety, frustration, and other negative emotional experiences that consume cognitive resources essential for comprehension and reinforce avoidance behaviours (Antonis, 2022; Cristofani et al., 2023; Rostami et al., 2024; Stein et al., 2024). At the same time, evidence suggests that higher trait emotional intelligence is associated with stronger reading self-concept, better academic performance, and lower anxiety, irrespective of diagnostic status (Donolato et al., 2024; Fiorilli et al., 2020; Quílez-Robres et al., 2023; Sánchez-Álvarez et al., 2020; Zaccoletti et al., 2020).

Recent evidence further shows that reading anxiety, general anxiety, and test anxiety are closely interrelated and negatively associated with word-reading fluency, text-reading fluency, and reading comprehension in students with reading difficulties (Fishstrom et al., 2024). In addition, comorbid inattention or hyperactivity has been linked to elevated anxiety in children with dyslexia, pointing to the complexity of the psychoemotional profile associated with reading difficulties (McArthur et al., 2024).

Taken together, these findings underscore that psychoemotional functioning should not be viewed as secondary to cognitive deficits, but rather as part of a broader, interacting system of learner characteristics that shapes reading development and academic adjustment. Person-centered evidence reinforces this view by showing that students with learning-related difficulties may follow different psychoemotional-motivational pathways, some marked by persistent vulnerability and others by relative strengths despite academic risk. They further suggest that interventions designed to strengthen emotional well-being, motivation, perceived competence, and self-regulatory resources may help mitigate the adverse effects of psychoemotional difficulties and, in turn, support more positive reading outcomes (Wei et al., 2020). In this respect, profile-based approaches may be especially informative, as they can help identify subgroups of students who share similar constellations of difficulties and strengths and may therefore benefit from more tailored forms of support.

#### 1.4.2. Students with Developmental Language Disorder

Compared with the literature on dyslexia, the psychoemotional profiles of students with DLD, particularly with respect to academic emotions and motivation, remain relatively underexplored, representing an important gap in the field (Donolato et al., 2024; Nachshon & Horowitz-Kraus, 2019). Nevertheless, existing research points to substantial socioemotional challenges in this population. Adolescents with DLD tend to exhibit greater difficulties in socioemotional functioning than their TD peers, and these vulnerabilities may persist across development (Arts et al., 2022; Burnley et al., 2023; Lloyd-Esenkaya et al., 2020). More specifically, children and adolescents with DLD have consistently been reported to experience more peer-related difficulties and higher rates of victimization (Forrest et al., 2021). Studies examining the lived experiences of individuals with DLD also emphasize the long-term impact of these difficulties on mental health and well-being across the lifespan (Wilmot et al., 2024).

Although some evidence suggests that adolescents with DLD may report levels of reading motivation comparable to those of TD peers despite lower comprehension performance, their underlying linguistic and cognitive difficulties continue to substantially affect reading outcomes (Tsang & Yeung, 2024). When faced with cognitively demanding texts, limited working memory capacity may become overloaded, hindering the integration of relevant linguistic information and increasing the likelihood of failure experiences and negative emotional responses (Wong et al., 2022). Emerging evidence further indicates that children with DLD may be at heightened risk for socioemotional difficulties compared with neurotypical peers (Yaşa & Çiyiltepe, 2024).

Overall, these findings suggest that reading comprehension may play a particularly important role in shaping psychoemotional patterns in children with DLD. However, this relationship remains insufficiently understood, especially from person-centered perspectives that could capture the heterogeneity of psychoemotional and cognitive profiles within this population.

### 1.5. The Present Study

Despite this evidence, there is limited research examining reading-specific psychoemotional–motivational profiles across TD, LD, and DLD groups. This gap is particularly important, as research indicates that children with neurodevelopmental disorders do not merely represent the lowest end of a broad range of emotions and motivation; rather, they display qualitatively different emotional experiences that need to be examined specifically (Donolato et al., 2024).

To address these gaps in the literature, the present study adopts a person-centered, cluster-analytic approach to identify and compare reading-specific psychoemotional profiles associated with reading comprehension across TD, LD, and DLD children. This data-driven approach captures patterns of co-occurring psychoemotional and motivational characteristics within individuals and allows for the identification of naturally occurring subgroups without imposing a priori assumptions, thereby moving beyond single-variable comparisons that may overlook intra- and inter-group heterogeneity (Zuppardo et al., 2023).

Specifically, the study aims to identify distinct psychoemotional–motivational profiles associated with RC and to examine how these profiles differ across TD, LD, and DLD groups. It further examines whether reading comprehension plays a differential role in psychoemotional profile differentiation across these developmental groups, with a potentially more central role in children with DLD. Given the exploratory, person-centered nature of the study, we formulated theoretically informed research questions rather than strict directional hypotheses.

To further guide the analysis, the study addressed the following research questions:

(RQ1) What distinct psychoemotional–motivational profiles associated with reading comprehension emerge within each diagnostic group (TD, LD, DLD)?

(RQ2) To what extent do emotional and motivational variables contribute to profile differentiation within each group?

(RQ3) Does reading comprehension play a more central role in profile differentiation in the DLD group than in the TD and LD groups?

By integrating cognitive and psychoemotional dimensions, this approach seeks to provide a more comprehensive understanding of the complex relationships among reading abilities, emotional regulation, and motivational processes. This approach is particularly valuable in distinguishing profiles that might otherwise be obscured by broad diagnostic categories (Jang & Conradi Smith, 2026; Jéldrez et al., 2025; Pasqualotto et al., 2024).

Overall, this approach may contribute to informing more targeted, emotion-sensitive educational practices by highlighting variability in students’ psychoemotional experiences.

## 2. Materials and Methods

### 2.1. Participants

The sample consisted of 83 third-grade elementary students (53% male) from 11 public schools in the prefecture of Attica, with a mean age of 107.45 months (8.9 years). Participants were classified into three groups: TD (*n* = 29), LD (*n* = 25), and DLD (*n* = 29).

Participants were classified into the TD, LD, and DLD groups based on existing formal diagnostic records provided by KEDASY, the public certified interdisciplinary assessment service responsible for evaluating students’ learning and psychosocial needs and confirmed through school documentation.

More than half of the parents had graduated from senior high school (26.5% of fathers, 25.3% of mothers). With regard to socio-economic status, most mothers were unemployed and engaged in household duties (28.9%), and most fathers (32.5%) were employed in office-based, administrative, professional, or managerial positions.

### 2.2. Procedure

Prior to data collection, consent for participation was obtained from school principals and teachers. Subsequently, written parental consent was requested, and parents were informed about the purpose of the study through a written notice prior to providing signed consent. Only students whose parents had provided consent were eligible to participate.

The administration of the research instruments followed a structured procedure. All questionnaires were distributed to students as a single package in a predetermined order. Standardized instructions were provided for each questionnaire, and additional clarifications were offered whenever requested.

To promote an unbiased response environment, students were explicitly informed that there were no right or wrong answers, that their responses would not be graded, and that anonymity would be ensured. To further minimize potential external influence, the classroom teacher was not present during the administration. The researcher read each item aloud to ensure consistent comprehension among participants. The total completion time was approximately two teaching hours. Students were also informed that they could withdraw from the process at any time if they wished.

Upon completion, all questionnaires were collected by the researcher for subsequent analysis. The study procedure complied with the “Code on Ethics and Good Practice” of the Research Ethics Committee of the National and Kapodistrian University of Athens (NKUA).

### 2.3. Measurements

For the purposes of this study, a set of questionnaires assessing psychoemotional factors, motivation, and learning was administered.

#### 2.3.1. Motivation for Reading Questionnaire—MRQ (Wigfield & Guthrie, 1997)

The MRQ is one of the most widely used instruments for assessing reading motivation (Mucherah & Yoder, 2008). The Greek adaptation of the questionnaire was developed by Dimitropoulou et al. (2016). The scale consists of 53 items rated on a 4-point Likert scale ranging from “very different from me” to “a lot like me.”

The MRQ includes 11 dimensions: (1) reading efficacy, (2) reading challenge, (3) work avoidance, (4) reading curiosity, (5) reading involvement, (6) importance of reading, (7) competition in reading, (8) reading recognition, (9) reading for grades, (10) social reasons for reading, and (11) reading compliance.

However, because several subscales demonstrated unsatisfactory levels of internal consistency, only the composite subscales representing intrinsic and extrinsic motivation—those showing satisfactory to very good Cronbach’s alpha values—were included in the present study. Intrinsic motivation was assessed through three dimensions: reading curiosity (e.g., “I read to learn new information about what interests me”), reading involvement (e.g., “I make pictures in my mind when I read a text”), and importance of reading (e.g., “It is important for me to be good at reading”).

Extrinsic motivation was assessed through the dimensions of reading recognition (e.g., “My parents often tell me that I am good at reading”), reading for grades (e.g., “I read to improve my grades”), and competition in reading (e.g., “I try to give more correct answers than my friends to text questions”).

Internal consistency was satisfactory across groups (α = 0.81–0.92). The use of these broader composite dimensions allowed us to retain the most psychometrically robust indicators of reading motivation while reducing measurement noise associated with weaker subscales.

#### 2.3.2. The Achievement Emotions Questionnaire—Elementary School (AEQ-ES) (Lichtenfeld et al., 2012)

The AEQ-ES scales were adapted from the Achievement Emotions Questionnaire (Pekrun et al., 2011). It was administered in groups to third-grade students to examine three emotions related to reading (pleasure, anxiety, and boredom) in three contexts (in the classroom, during homework, and in reading assessment).

Students were asked to rate their level of agreement on a five-point scale with each of the 28 statements in the questionnaire.

Indicative examples include: “Reading is enjoyable for me,” “Reading assignments bore me terribly,” and “When my teacher tests me on reading, I get very anxious.”

Due to their young age and to facilitate completion, the response options were accompanied by an image of a child’s face matching the participant’s gender (where 1 = I strongly disagree—no smile and 5 = I strongly agree—very big smile).

Scores we calculated scores of positive and negative emotions across the three conditions. Internal consistency was satisfactory across groups (α = 0.82–0.94).

#### 2.3.3. Reading Comprehension Battery (Chrysochoou, 2006)

Reading comprehension was assessed using a battery developed by (Chrysochoou, 2006; see also Chrysochoou & Bablekou, 2011; Chrysochoou et al., 2011, 2018), which was developed in the absence of standardized tests in Greek and provides measures of higher-order comprehension skills.

Participants were presented with 5 written stories—one of which was used for practice—accompanied by 10 open-ended questions tapping 4 higher-order comprehension skills: generation of necessary and elaborative inferences, simile comprehension, and comprehension control.

Stories and some of the questions were drawn from Oakhill (1984) and Cain and Oakhill (1999). Additional questions were added, and the stories were adapted for use with Greek children.

Students were asked to read each text within a given time frame and then answer the questions on the next page briefly, without turning the page and looking at the text.

Students were given eight minutes for each text, after which the researcher instructed them to move on to the next text.

The order of the stories differed across students.

Internal consistency was satisfactory across groups (α = 0.81–0.91).

### 2.4. Statistical Analyses

The use of cluster analysis in the present study is grounded in a person-centered methodological framework, which aims to identify meaningful subgroups based on patterns of co-occurring variables rather than isolated effects (Meyer et al., 2013).

Although subgroup sample sizes were relatively modest, each analysis included a limited number of variables, resulting in an acceptable case-to-variable ratio according to commonly cited guidelines for exploratory cluster analysis (e.g., 5–10 cases per variable; Field, 2013; Henry et al., 2005).

Furthermore, comparable sample sizes have been used in prior person-centered research, particularly in studies involving clinical or hard-to-access populations (Howard & Hoffman, 2018). Cluster analyses were conducted separately within each diagnostic group to preserve developmental and clinical specificity.

Given the exploratory nature of the study, emphasis was placed on the interpretability and stability of the identified cluster solutions (Spurk et al., 2020).

Hierarchical cluster analyses using Ward’s (1963) minimum-variance method and squared Euclidean distance were conducted separately for each group. The optimal number of clusters was determined based on the agglomeration schedule and dendrogram inspection (Borgen & Barnett, 1987). A two-cluster solution was retained and subsequently refined using k-means clustering to enhance classification accuracy.

Cluster solutions were evaluated based on multiple established and converging criteria, including inspection of the agglomeration schedule, dendrogram structure, and subsequent k-means refinement. In line with recommendations for exploratory clustering approaches, particular emphasis was placed on interpretability and parsimony of the identified profiles (Hair et al., 2019; Hennig & Meila, 2015).

Given the relatively small subgroup sizes, cluster validity was primarily assessed through these complementary procedures rather than relying on a single quantitative index.

Cluster analyses were conducted on standardized (z-score) variables, including age (in months), reading comprehension, positive and negative academic emotions, and intrinsic and extrinsic motivation.

The ANOVA statistics provided in the k-means output were used descriptively to aid interpretation of cluster differentiation and were not treated as inferential tests.

To formally examine differences between clusters, additional one-way ANOVAs were conducted within each group using cluster membership as the independent variable. Effect sizes (partial η^2^) were calculated. Assumptions of normality and homogeneity of variance were examined prior to analysis. Where minor deviations were observed, results were interpreted cautiously.

Given the relatively small sample size, bootstrapping procedures (2000 resamples, bias-corrected 95% confidence intervals) were applied to obtain robust estimates.

## 3. Results

Cluster analyses were conducted separately within each diagnostic group (TD, LD, and DLD) to identify reading-specific psychoemotional patterns. A two-cluster solution was retained for all groups based on the agglomeration schedule and dendrogram inspection.

**Figure 1:**
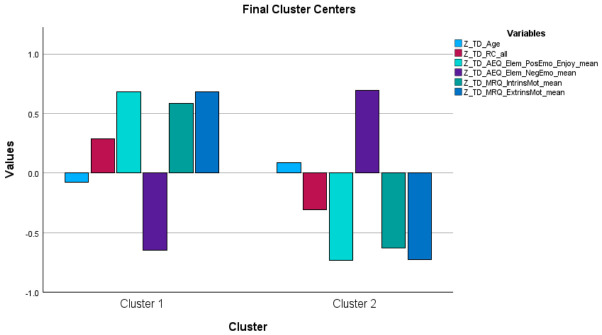
The two clusters identified in the k-means cluster analysis performed in the Typically Developing group.

**Figure 2:**
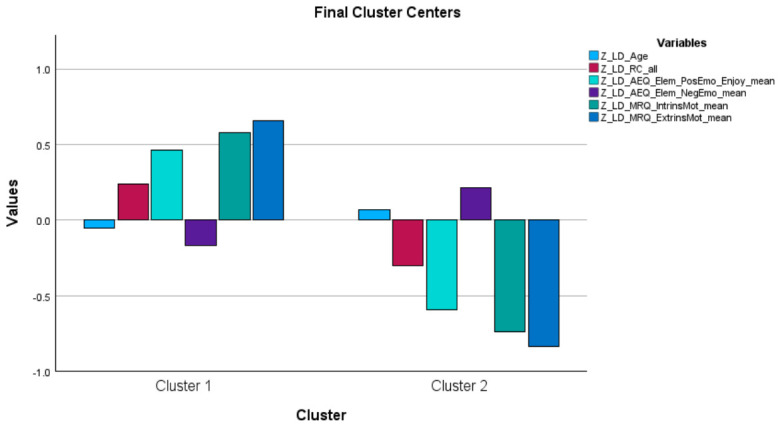
The two clusters identified in the k-means cluster analysis performed in the Learning Disabilities group.

**Figure 3:**
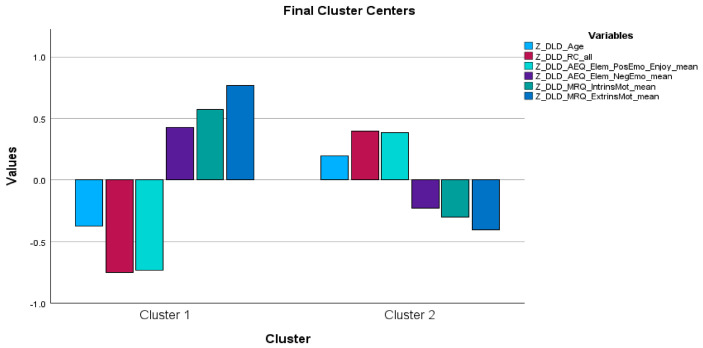
The two clusters identified in the k-means cluster analysis performed in the DLD group.

The results of the k-means cluster analyses within each group are presented below (see Table 1, Table 2 and Table 3 and Figure 1, Figure 2 and Figure 3).

The ANOVA statistics reported in the tables are derived from the k-means procedure and are presented for descriptive purposes only, as indicators of the variables contributing to cluster differentiation, and are not treated as inferential tests.

To formally examine differences between clusters, additional one-way ANOVAs were conducted within each group using cluster membership as the independent variable. Effect sizes (partial η^2^) were calculated, and assumptions of normality and homogeneity of variance were examined.

Given the relatively small sample size, bootstrapping procedures (2000 resamples, bias-corrected 95% confidence intervals) were applied to obtain robust estimates. Detailed ANOVA results, including effect sizes, are presented in Table 1, Table 2 and Table 3.

It should be noted that, given the relatively small subgroup sizes, the cluster solutions should be interpreted as exploratory and hypothesis-informed, representing preliminary patterns rather than stable profiles.

In line with the study’s research questions, the results below focus on (a) the psychoemotional–motivational profiles emerging within each group, (b) the variables contributing to profile differentiation, and (c) the extent to which reading comprehension contributed to cluster separation across groups.

### 3.1. Typically Developing Children

The k-means cluster analysis conducted for the typically developing children (N = 29 third graders) revealed two reading-specific psychoemotional profiles: (a) a profile (N = 15) characterized by higher positive emotion, lower negative emotion, and higher intrinsic and extrinsic reading-specific motivation, and (b) a profile (N = 14) with the opposite characteristics.

The two profiles did not differ in terms of age (in months) or reading comprehension performance.

Consistent with the ANOVA results, cluster differentiation in the TD group was primarily driven by emotional and motivational variables, whereas reading comprehension did not significantly differentiate the clusters (see Table 1).

The number of boys and girls also did not differ between clusters (χ^2^ = 1.71, df = 1, *p* = 0.191; six boys and nine girls in the first cluster, and nine boys and five girls in the second cluster).

Overall, the two clusters in the TD group may be interpreted as reflecting more versus less adaptive psychoemotional–motivational profiles, rather than differences in cognitive performance.

Thus, in the TD group, the results primarily address RQ1 and RQ2, whereas reading comprehension did not contribute substantially to profile differentiation.

### 3.2. Children with Learning Disabilities

The k-means cluster analysis conducted for identifying reading-specific psychoemotional profiles among children with learning disabilities (N = 25 third graders) revealed a partially similar pattern: (a) a profile (N = 14), characterized by higher positive emotion, lower negative emotion, and higher intrinsic and extrinsic reading-specific motivation, and (b) a profile (N = 11) with the opposite characteristics.

The two profiles did not differ in terms of age (in months) or reading comprehension performance.

ANOVA results indicated that cluster differentiation was primarily driven by intrinsic and extrinsic motivation, as well as positive emotion, whereas negative emotion and reading comprehension did not significantly distinguish the clusters (see Table 2).

The number of boys and girls also did not differ between clusters (χ^2^ = 0.47, df = 1, *p* = 0.495; seven boys and seven girls in the first cluster, and seven boys and four girls in the second cluster).

Overall, the clusters in the LD group reflect differences in motivational and positive emotional engagement, with less consistent differentiation in negative emotional responses.

Similarly, in the LD group, the findings primarily address RQ1 and RQ2, whereas reading comprehension did not emerge as a central differentiating variable.

### 3.3. Children with Developmental Language Disorder

Finally, the k-means cluster analysis conducted for children with DLD (N = 29 third graders) revealed two reading-specific psychoemotional profiles: (a) a profile (N = 10) characterized by lower reading comprehension performance and positive emotion, but higher intrinsic and extrinsic motivation levels, and (b) a profile (N = 19) with the opposite characteristics.

The two profiles did not differ in terms of age or negative emotion.

The number of boys and girls did not differ between clusters (χ^2^ = 0.18, df = 1, *p* = 0.893; five boys and five girls in the first cluster, and ten boys and nine girls in the second cluster).

Overall, the clusters in the DLD group reflect a more integrated psychoemotional–cognitive differentiation, with both emotional–motivational variables and reading comprehension contributing to cluster separation.

In this respect, the DLD findings address all three research questions and provide the strongest support for RQ3, namely that reading comprehension appeared to play a more central role in profile differentiation in this group.

## 4. Discussion

The aim of the present study was to examine psychoemotional profiles associated with reading comprehension in third-grade Greek students with TD, LD, particularly dyslexia, and DLD. Specifically, the study examined how academic emotions (positive and negative) and reading-specific motivation (intrinsic and extrinsic) relate to reading comprehension performance. Using a person-centered approach, the study sought to identify distinct psychoemotional profiles that may tentatively inform more targeted, emotion-sensitive educational practices. In this way, the study contributes to a better understanding of the role of psychoemotional processes in reading development, rather than establishing causal intervention pathways.

Overall, the findings provided partial support for the research questions. Distinct psychoemotional–motivational profiles emerged across all three groups, and emotional and motivational variables contributed to profile differentiation in each group, whereas reading comprehension appeared to play a more central differentiating role only within the DLD group. This pattern is elaborated further below.

A particularly noteworthy finding concerns the psychoemotional profile identified in the DLD group, characterized by lower reading comprehension, higher negative emotions, and relatively high levels of motivation. This configuration is especially important, as it diverges from patterns typically reported in the dyslexia literature, where reading difficulties are more often associated with lower motivation. From the perspective of control–value theory and self-determination theory, this pattern may tentatively suggest that motivational engagement can be maintained despite low perceived competence? Further empirical investigation is required before firm conclusions can be drawn.

Among TD third graders, two distinct psychoemotional patterns emerged, differentiated primarily by emotional and motivational characteristics, with no significant differences in reading comprehension performance, in line with the ANOVA results. Specifically, the first profile was characterized by higher positive academic emotions, lower negative academic emotions and higher intrinsic and extrinsic reading motivation than the second. This pattern indicates that, at this developmental phase, variations in emotional and motivational orientations may not necessarily translate into measurable differences in reading comprehension. This is consistent with RQ2, according to which emotional and motivational variables would contribute to profile differentiation, while reading comprehension would not necessarily play a central role in all groups.

Although previous research has suggested that intrinsic motivation supports reading comprehension (Fang, 2024; Toste et al., 2020; Troyer et al., 2019), the present findings indicate that different psychoemotional orientations may coexist with similar levels of reading comprehension at this age.

Interpretations involving compensatory strategies, increased effort or other buffering mechanisms should remain tentative, as these processes were not directly assessed. One possible explanation is that some students may maintain comparable performance despite less adaptive psychoemotional patterns, although this remains speculative.

From a theoretical perspective, this pattern may be cautiously considered in light of Control-Value Theory, according to which certain activating negative emotions, such as anxiety, may temporarily sustain engagement (Zaccoletti et al., 2020). However, because emotional processes were not examined longitudinally, such interpretations should be treated with caution.

Thus, although students in the less adaptive profile may attain comparable levels of reading comprehension, they may do so under different psychoemotional conditions, with possible implications for longer-term engagement. If such patterns persist over time, they may potentially be associated with a greater risk of later academic disengagement or emotional exhaustion; however, this remains to be tested longitudinally.

A partially similar pattern was observed in the LD group, largely reflecting dyslexia-related characteristics in this developmental stage. The two profiles differed primarily in psychoemotional and motivational characteristics, with the first profile exhibiting higher positive academic emotions, lower negative academic emotions, and higher intrinsic and extrinsic reading-specific motivation than the second, while again, no significant differences were found in reading comprehension or age.

These findings support the presence of psychoemotional heterogeneity within the LD group, without corresponding differences in reading comprehension performance. This pattern similarly provides support for RQ1 and RQ2, while suggesting that reading comprehension was not a central differentiating factor in this group. Although previous literature has consistently shown that students with LD tend to report lower motivation and higher negative affect than their TD peers, the present findings extend this evidence by suggesting that such difficulties are not uniformly distributed within the LD group. (Bonuomo et al., 2023; Chiappedi & Baschenis, 2014; Cristofani et al., 2023; Donolato et al., 2024; Iaia et al., 2024; Mammarella et al., 2016; Martín-Ruiz et al., 2023; Matteucci et al., 2019; Polychroni et al., 2024; Sideridis, 2005, 2006; Sideridis et al., 2006, 2013, 2016; Visser et al., 2020; Zaccoletti et al., 2020).

In particular, a subgroup of students with LD may display relatively more adaptive psychoemotional patterns toward reading, although the mechanisms underlying this pattern were not directly assessed.

Interpretations related to resilience or buffering mechanisms should therefore remain tentative, especially given evidence that persistent reading difficulties are often associated with heightened negative emotions and reduced motivation (Fishstrom et al., 2024; Mano et al., 2017; Rostami et al., 2024; Zaccoletti et al., 2020).

At the same time, the presence of the less adaptive profile—more consistent with the psychoemotional difficulties commonly associated with dyslexia—highlights the substantial heterogeneity within this diagnostic group and underscores the limitations of relying solely on traditional group comparisons. Importantly, this pattern closely resembled that observed in the TD group, where psychoemotional differences between profiles likewise did not correspond to measurable differences in reading comprehension.

Overall, the similarity between the TD and LD groups contrasts with the pattern observed in the DLD group, which may suggest that psychoemotional variability may reflect internal psychological differences that do not necessarily manifest in immediate performance outcomes.

The cluster analysis for children with DLD revealed a more clearly differentiated pattern of two reading-specific psychoemotional profiles than that observed in the TD and LD groups. Profile 1 was characterized by lower reading comprehension, lower positive academic emotions, higher negative academic emotions, and higher intrinsic and extrinsic reading-specific motivation levels. On the other hand, Profile 2 displayed higher reading comprehension performance, more positive feelings about reading, lower negative emotions, and lower intrinsic and extrinsic motivation levels. Unlike the other two groups, the profiles within the DLD group differed significantly in reading comprehension, suggesting that reading comprehension may be a central factor in cluster differentiation and directly addressing RQ3.

The combination of lower reading comprehension and elevated negative emotions with higher motivation in DLD Profile 1 represents a notable pattern. Unlike patterns typically reported in dyslexia, where academic difficulties are often accompanied by reduced motivation (Donolato et al., 2024; Polychroni et al., 2024; Wei et al., 2020), the present findings may suggest that some students with DLD may remain motivated despite experiencing comprehension difficulties and negative emotional states such as frustration or failure (Mano et al., 2017; Nachshon & Horowitz-Kraus, 2019; Wong et al., 2022).

One possible interpretation is that these students continue to invest motivational resources despite limited reading outcomes, which may suggest that cognitive constraints may generate emotional distress without necessarily reducing motivational engagement (Camacho-Morles et al., 2021; Lam et al., 2024; Mano et al., 2017; St Clair et al., 2019; Zaccoletti et al., 2020).

Although this pattern may tentatively be viewed in light of Self-Determination Theory and sociocultural perspectives, according to which motivation can be sustained despite challenges in competence through contextual or relational support (Fang, 2024; Wigfield & Koenka, 2020), such interpretations should remain tentative because the underlying mechanisms were not directly examined.

By contrast, Profile 2 combined higher reading comprehension and more positive emotions with lower motivation, a pattern that is less readily interpretable and warrants further investigation.

Overall, the divergence between the two DLD Profiles may suggest that reading comprehension in this group may be more closely linked to psychoemotional differences than in the TD and LD groups, where psychoemotional variability did not correspond to measurable differences in reading comprehension. These findings suggest that, although psychoemotional factors contributed to profile differentiation across all three groups, reading comprehension may play a more central differentiating role specifically in the DLD group.

This pattern is broadly consistent with Control-Value Theory (Pekrun, 2022), according to which heightened negative emotions may increase cognitive load and interfere with higher-order comprehension processes (Burnley et al., 2023; St Clair et al., 2019; Zaccoletti et al., 2020).

Importantly, motivation appeared as part of broader psychoemotional profiles rather than as a direct and consistent predictor of reading comprehension performance, except in the DLD group, where it co-occurred with differences in comprehension.

A further important finding across all three groups was that age did not contribute to profile formation, as the two clusters within each group did not differ significantly in age. This indicates that the observed psychoemotional patterns were not driven by age-related variation within this developmental phase.

Positive academic emotions significantly differentiated profile membership in all three groups, indicating that positive affect was a consistent component of psychoemotional variability; however, its association with reading comprehension was evident only in the DLD group.

Positive emotions may be associated with greater engagement in reading, which has been linked to improved comprehension processes (Oatley, 2012; Pekrun, 2022; Prinz-Weiß et al., 2023). However, this relationship was not consistently reflected in the present findings.

Interestingly, our study found that negative affect contributed to profile differentiation primarily in the TD group, which may suggest that negative emotional responses may play a stronger role in shaping engagement or strategy use among typically developing students.

At the same time, our analysis also revealed that motivation (both intrinsic and extrinsic combined) contributed to profile formation across all groups, not as opposing dimensions but as combined motivational patterns, indicating that profile differentiation was based on constellations of motivational orientations rather than on a simple intrinsic–extrinsic distinction (Corpus & Wormington, 2014).

This supports the view that motivation and emotions operate as interrelated components of the psychoemotional experience of reading rather than as isolated predictors of reading outcomes. Consistent with person-centered work (Miller-Cotto & Byrnes, 2026; Nyman et al., 2025; Wang et al., 2020, 2022), the present findings further suggest that intrinsic and extrinsic motivation may co-occur and jointly shape reading-related behavior.

Overall, these findings underscore the importance of considering psychoemotional variability in understanding reading comprehension across both typical and atypical populations. The person-centered approach adopted in this study highlights the heterogeneity of psychoemotional profiles within TD, LD, and DLD groups—an aspect that has received limited attention in reading comprehension research—and may suggest that the role of reading comprehension in profile differentiation may differ across developmental groups, with a potentially central role in DLD.

### 4.1. Implications for Educational Practice

The results of this study may have potential implications for the educational context, particularly for third-grade Greek students. Third grade is a pivotal developmental stage, representing a critical transition where children are expected to shift from mastering basic decoding skills to utilizing reading as a primary tool for learning across subjects (van Zeijts et al., 2024). At this stage of development, children’s cognitive capacities are still maturing, making them potentially susceptible to the effects of psychoemotional distress on higher-order comprehension processes.

The identification of distinct psychoemotional profiles within each group, particularly the DLD group, may suggest that uniform instructional approaches may not always adequately capture individual differences within the Greek educational system. The findings may indicate the potential value of individualized, emotion-sensitive approaches alongside cognitive-based strategies.

More specifically, interventions for all three groups (TD, LD, DLD) may benefit from moving beyond a sole focus on cognitive skill development. The present findings may highlight that academic difficulties are closely intertwined with students’ emotional well-being. In this respect, the Greek educational system may potentially benefit from a more holistic educational approach that integrates mental health support within schools, potentially through school psychologists or specialized support staff, to address the psychoemotional needs identified in these students, including the social-emotional challenges associated with DLD (Forrest et al., 2021).

For students with LD and TD who display lower levels of positive emotions, higher levels of negative emotions, and reduced intrinsic and extrinsic reading-specific motivation, instructional strategies could tentatively prioritize the enhancement of intrinsic motivation. This may be achieved through the provision of differentiated reading materials aligned with students’ interests, learning profiles, and readiness levels, thereby potentially helping to reduce negative emotional experiences and promote more positive engagement with reading.

For DLD students, the observed discrepancy between high motivation and lower reading comprehension alongside elevated negative emotions may point to the need for a more targeted approach. This could tentatively involve explicit strategy instruction to reduce cognitive load and improve comprehension, combined with emotional regulation techniques to manage negative emotions, thereby potentially capitalizing on their existing motivation (Zaccoletti et al., 2020), although the effectiveness of such approaches remains to be empirically tested.

For DLD Profile 1, educators may consider leveraging students’ relatively high levels of motivation. This could involve providing opportunities for successful reading experiences, offering choices in reading materials, and explicitly teaching metacognitive strategies to support a sense of competence (Fang, 2024). Such approaches may potentially help reduce the risk of motivational decline observed in students with persistent academic difficulties.

In addition, early and systematic screening for psychoemotional risk factors (e.g., reading anxiety, low reading self-concept, specific motivational patterns), alongside traditional reading assessments, especially for students showing early signs of LD or DLD, may be beneficial. The early identification of profiles such as DLD Profile 1 may allow educators to implement timely support, which may potentially prevent the escalation of academic and emotional difficulties (Yaşa & Çiyiltepe, 2024).

Finally, an important prerequisite may be teacher training in recognizing students’ psychoemotional differences and adapting their instructional approaches accordingly. This includes understanding that high motivation in a struggling DLD student may reflect persistent effort rather than reduced need for support, and that addressing emotional factors may be important for supporting learning outcomes.

### 4.2. Limitations and Future Directions

This study has several methodological limitations that should be considered when interpreting the findings. The term “profile” is used descriptively to refer to cluster-based groupings. However, cluster analysis assigns individuals deterministically to groups, may constitute a methodological limitation. Future research using model-based approaches, such as latent profile analysis, may provide a more nuanced understanding by incorporating classification uncertainty.

Moreover, the sample sizes for each group were relatively small, which may limit the generalizability of the identified patterns and the detection of more nuanced subgroups. Because data were collected at a single point in time, it is not possible to determine how these patterns emerge, change, or stabilize across developmental stages. In addition, this design does not permit causal inferences regarding the directionality of the relationships between psychoemotional factors and reading comprehension.

Thus, it remains unclear whether psychoemotional characteristics influence reading comprehension outcomes, whether reading difficulties contribute to psychoemotional distress, or whether these relationships are reciprocal or shaped by additional factors over time. Additionally, the study was conducted within a specific Greek educational context. Educational practices and curricular demands related to reading and emotional expression may vary across contexts, potentially influencing both psychoemotional patterns and reading motivation.

Furthermore, the use of self-report questionnaires introduces methodological limitations. Such measures may be influenced by social desirability bias, particularly in younger populations, which may affect the accuracy of reported emotional and motivational experiences. Consequently, the present findings should be interpreted with caution, as they reflect perceived rather than directly observed processes.

Longitudinal studies are needed to examine the stability and developmental trajectories of psychoemotional profiles over time, particularly in children with DLD. Larger samples would allow for the identification of more differentiated patterns. Further research examining the nature of motivation in DLD (e.g., intrinsic vs. extrinsic; mastery vs. performance orientations) may help inform more targeted intervention approaches.

Another important direction concerns the long-term impact of psychoemotional patterns on academic achievement and well-being, particularly through methodologies capable of capturing the interplay between language ability, reading self-concept, social anxiety, and reading comprehension. The incorporation of mixed-methods designs may further enhance this line of research. Combining quantitative profiling techniques with qualitative approaches (e.g., interviews, classroom observations) could provide deeper insight into students’ emotional and motivational experiences and help clarify the mechanisms underlying profile membership.

## Figures and Tables

**Table 1:** Cluster Centers and ANOVA Results for the Typically Developing Group.

Measures	Cluster 1 (Center)	Cluster 2 (Center)	Inferential ANOVA
Age (months)	−0.08	0.09	*F*(1,27) = 0.19, *p* = 0.661, η^2^_p_ = 0.01
Reading comprehension	0.29	−0.31	*F*(1,27) = 2.73, *p* = 0.102, η^2^_p_ = 0.09
Positive emotion	0.68	−0.73	*F*(1,27) = 28.86, *p* < 0.001, η^2^_p_ = 0.52
Negative emotion	−0.65	0.69	*F*(1,27) = 23.23, *p* = 0.003, η^2^_p_ = 0.46
Reading-Intrinsic motivation	0.59	−0.63	*F*(1,27) = 16.76, *p* = 0.001, η^2^_p_ = 0.38
Reading-Extrinsic motivation	0.68	−0.73	*F*(1,27) = 28.65, *p* < 0.001, η^2^_p_ = 0.52

*Note.* Inferential ANOVA results (*F*, *p*, η^2^_p_) are reported. Bootstrapping procedures (2000 resamples) were applied.

**Table 2:** Cluster Centers and ANOVA Results for the Learning Disabilities Group.

Measures	Cluster 1 (Center)	Cluster 2 (Center)	Inferential ANOVA
Age (months)	−0.05	0.07	*F*(1,23) = 0.09, *p* = 0.768, η^2^_p_ = 0.00
Reading comprehension	0.24	−0.30	*F*(1,23) = 1.89, *p* = 0.182, η^2^_p_ = 0.08
Positive emotion	0.46	−0.59	*F*(1,23) = 9.23, *p* = 0.006, η^2^_p_ = 0.29
Negative emotion	−0.17	0.22	*F*(1,23) = 0.91, *p* = 0.349, η^2^_p_ = 0.04
Reading-Intrinsic motivation	0.58	−0.74	*F*(1,23) = 18.53, *p* < 0.001, η^2^_p_ = 0.45
Reading-Extrinsic motivation	0.66	−0.83	*F*(1,23) = 30.56, *p* < 0.001, η^2^_p_ = 0.57

*Note.* Inferential ANOVA results (*F*, *p*, η^2^_p_) are reported. Bootstrapping procedures (2000 resamples) were applied.

**Table 3:** Cluster Centers and ANOVA Results for the Developmental Language Disorder Group.

Measures	Cluster 1 (Center)	Cluster 2 (Center)	Inferential ANOVA
Age (months)	−0.37	0.20	*F*(1,27) = 2.21, *p* = 0.149, η^2^_p_ = 0.08
Reading comprehension	−0.75	0.40	*F*(1,27) = 12.03, *p* = 0.002, η^2^_p_ = 0.31
Positive emotion	−0.73	0.38	*F*(1,27) = 11.08, *p* = 0.003, η^2^_p_ = 0.29
Negative emotion	0.43	−0.23	*F*(1,27) = 3.02, *p* = 0.094, η^2^_p_ = 0.10
Reading-Intrinsic motivation	0.57	−0.30	*F*(1,27) = 5.91, *p* = 0.022, η^2^_p_ = 0.18
Reading-Extrinsic motivation	0.77	−0.40	*F*(1,27) = 12.86, *p* < 0.001, η^2^_p_ = 0.32

*Note.* Inferential ANOVA results (*F*, *p*, η^2^_p_) are reported. Bootstrapping procedures (2000 resamples) were applied.

## Data Availability

Data available in a publicly accessible repository https://osf.io/7t5bw/overview?view_only=b6287a1f945c4bc7bd8bb2bd9e16bfe9 (accessed on 10 March 2026).
